# Frequency of Intradialytic Hypertension Using Kidney Disease: Improving Global Outcomes (KDIGO) Suggested Definition in a Single Hemodialysis Centre in Pakistan

**DOI:** 10.7759/cureus.33104

**Published:** 2022-12-29

**Authors:** Farah Mujtaba, Ruqaya Qureshi, Murtaza Dhrolia, Kiran Nasir, Aasim Ahmad

**Affiliations:** 1 Nephrology, The Kidney Centre Post Graduate Training Institute, Karachi, PAK

**Keywords:** kdigo, intradialytic hypertension, hypertension, hemodialysis, end stage kidney disease

## Abstract

Objectives: To estimate the frequency of intradialytic hypertension (IDH) in our centre as per the definition suggested by Kidney Disease: Improving Global Outcomes (KDIGO).

Methods: A cross-sectional study was conducted at the dialysis department of The Kidney Centre Post Graduate Training Institute (PGTI) Karachi, Pakistan from August 2021 to October 2021 among 263 end-stage kidney disease (ESKD) patients on maintenance hemodialysis (MHD) aged ≥ 18 years of both genders. The study outcome was the frequency of IDH as per the latest KDIGO suggested definition i.e., systolic blood pressure (SBP) rise of > 10 mm Hg from pre- to post-dialysis within the hypertensive range in at least four out of six consecutive dialysis treatments. Frequencies (%) and mean (±SD) were calculated for categorical and continuous variables respectively, using SPSS version 21 (IBM Corp., Armonk, NY, USA).

Results: Among 263 patients, the mean age was 51.02 (±14.1) years and 56.3% were males. Around 30.8% of patients were dialysis-dependent for 1.1 to three years. The most common comorbidity was hypertension (88.6%). Standard dialysate calcium of 3mEq/l was received by 91.6% of study participants. About 78.7% of patients were using antihypertensive(s), out of which 85.5% were compliant and 37.6% were using a single antihypertensive. The most common antihypertensive in use was beta-blockers (78.3%). Around 16% of patients were found to have IDH. Age of the patients was significantly associated with IDH (p=0.038). The majority of the patients with IDH were those who were taking anti-hypertension medications as compared to the patients who were not taking them (p <0.004). Interdialytic weight gain was not a significant predictor for IDH.

Conclusion: The frequency of IDH was 16% according to the latest suggested KDIGO definition. This is much lower than regional and global estimates according to earlier definitions. There is a dire need to establish a standardized definition of IDH in guidelines to diagnose, manage and compare data. Also, the association of IDH with fluid overload is not found in our study which emphasizes the need to evaluate other causative factors.

## Introduction

Among all comorbidities, hypertension (HTN) is almost universally present in end-stage kidney disease (ESKD) patients on maintenance hemodialysis (MHD). The reason for such high prevalence is a combination of different pathophysiological mechanisms, including renin-angiotensin-aldosterone system, endothelial dysfunction, arterial stiffness, and increased sympathetic nervous system activity [1]. Another factor that adds to this difficulty is the development of extracellular volume expansion in ESKD patients [1]. This requires pharmacological therapies and fluid management for blood pressure (BP) control in ESKD patients.

The most challenging part of the management of ESKD patients is the frequent BP change that occurs in between and during hemodialysis treatment. The BP abnormalities during dialysis treatment include intradialytic hypotension and intradialytic hypertension (IDH). 

Up till now, there is no consensus on the definition of IDH. Arbitrary clinical definitions include BP rise of any degree during the second or third intradialytic hour, systolic blood pressure (SBP) rise >15 mm Hg within or immediately post-dialysis, SBP rise >10 mm Hg from pre- to post-dialysis or rising intradialytic BP that is unresponsive to volume removal [2]. The Kidney Disease: Improving Global Outcomes (KDIGO) suggested definition is SBP rise >10 mm Hg from pre- to post-dialysis in the hypertensive range in at least four out of six consecutive dialysis treatments [2].

The proposed pathophysiology of IDH involves extracellular volume overload and peripheral vasoconstriction [1]. Extracellular volume overload is a consistent finding in patients with intradialytic hypertension [3,4]. Some studies have also implicated the etiology of an increase in peripheral vascular resistance causing intradialytic hypertension [3,5]. These studies show that the vasoconstrictive component is the likely cause of the increase in BP during dialysis, while others fail to support this hypothesis and propose the imbalance of vasodilators as a probable explanation [6]. Besides these causes, there are several other risk factors like dialysate sodium, dialysate calcium, dialysate temperature, sodium profiling and ultrafiltration rate that affect BP during dialysis [7].

The frequency of IDH has shown variability over time and across countries due to the involvement of multiple risk factors, as studied by Park et al. [7]. Research conducted in the 1990s shows the frequency of IDH as 5% to 15% [8]. Recent studies show a frequency of 22.3% in the USA [9], 40.5% in the West African country Guinea [10], and 34.1% in Indonesia [11]. The neighboring country India has a frequency of 34.51% [12]. To our knowledge, no study has been conducted yet in Pakistan to find the frequency of IDH where the chronic kidney disease (CKD) burden is huge. 

Due to a lack of consensus on the definition of IDH, the current data available on the frequency of IDH is based on different definitions. This brings difficulty in comparing data, assessment of risk factors, and making newer strategies for its management. Intradialytic hypertension carries an exceedingly high risk of morbidity and mortality [13,14]; therefore, there is a dire need to reach a unified consensus on its definition.

## Materials and methods

Study design, setting, and participants

A hospital-based cross-sectional study was carried out at the dialysis department of The Kidney Centre Post Graduate Training Institute (TKC PGTI) from August 2021 to October 2021. A non-probability consecutive sampling technique was used. The total number of registered patients at the dialysis hall was 419 out of which 270 fulfilled our inclusion and exclusion criteria. All patients 18 years of age and above, irrespective of gender, on maintenance hemodialysis thrice weekly for more than three months, with apparently stable dry weight were included in our study. Patients on maintenance hemodialysis for less than three months and whose dry weight was currently being adjusted were excluded. 

Sample size estimation

The sample size calculation was done using the Open Epi Version 3.01 and came out to be 254 based on the frequency of intradialytic hypertension i.e. 34.5% [12], considering a 1% margin of error, 95% confidence level using 419 population size.

Procedure

Informed written consent in English/Urdu was taken from eligible candidates. A total of 13 dialysis sessions for each patient were studied. 

Statistical analysis

Data was entered and analyzed on IBM SPSS version 26 (IBM Corp., Armonk, NY, USA). Cleaning and coding of the data were done prior to analysis. Continuous data like age, BP, and weight were expressed in Mean±Std, while the frequency with percentage was calculated for categorical data like gender and comorbid. The normality of continuous variables was checked by the Shapiro-Wilk test. To observe any mean difference between pre- and post-hemodialysis BP and weight of the same patients paired t-test was applied in normally distributed data, while the Wilcoxon sign rank test was used on skewed parameters. To see any mean difference between the two groups, the student t-test or Mann-Whitney U test was applied according to the normality of the data. Association between the variables was accessed by Chi-square or Fisher exact test as appropriate. The significant variables of univariate analysis were kept in the multivariable binary logistic regression model and an odds ratio with a 95% confidence interval was obtained. A p-value of ≤0.05 was considered significant. 

Ethical consideration

The Kidney Centre Ethical Review Committee provided ethical approval for the study (Reference No. 112-NEPH-122020).

## Results

Of around 419 patients registered at TKC PGTI Dialysis Hall, 32 expired during the study period, 13 transferred to another facility and three patients underwent a transplant. Of the remaining 371 patients, 270 fulfilled our inclusion criteria. Two hundred sixty-three patients agreed to participate with a response rate of 97%. 

Out of 263 patients, 148 (56.3%) were male while 115 (43.7%) were female. The mean age was 51.02 ± 14.1 years, median was 51 years with a minimum of 20 and a maximum of 90 years. The most prevalent comorbidity was HTN (233; 88.6%) in our patients followed by DM (96; 36.5%). The majority (81; 30.8%) were dialysis dependent between 1.1 to three years followed by 67 (25.5%) being on dialysis between 5.1 to 10 years. The most common access for dialysis was arteriovenous fistula (AVF) in 253 (96.2%) patients. Arteriovenous graft (AVG) was found in five (1.9%) patients and another five (1.9%) had a tunneled catheter with the most common site being subclavian in three patients. Dialysate calcium of 3mEq/L was received by 241 (91.6%) of the study participants, while the remaining had dialysate calcium of 2mEq/L (Table 1). Standard dialysate sodium of 135mEq/L was used in all participants.

**Table 1:** Baseline demographic and clinical characteristics of the patients (n= 263)

Parameters		N (%)/Mean±Std
Age		51.02 ± 14.1
Gender	Male	148(56.3)
	Female	115(43.7)
Comorbid	Hypertension	233(88.6)
	Diabetes mellitus	96(36.5)
	Ischemic heart disease	57(21.7)
	Cardiomyopathy	54(20.5)
	Cerebrovascular disease	24(9.1)
	Hepatitis B +	18(6.8)
	Hepatitis c +	15(5.7)
	Transplant rejection	7(2.7)
Current Angio access	Arteriovenous fistula	253(96.2)
	Arteriovenous graft	5(1.9)
	Tunneled catheter	5(1.9)
Site of catheter (n=5)	Internal jugular	2(40)
	Sub-clavian	3(60)
Dialysate calcium(mEq/L)	2	22(8.4)
	3	241(91.6)
Dialysis vintage	3-6 months	14(5.3)
	6.1 - 12 months	26(9.9)
	1.1 - 3 years	81(30.8)
	3.1 - 5 years	46(17.5)
	5.1 - 10 years	67(25.5)
	> 10 years	29(11)

Out of the total, 207 (78.7%) patients were taking anti-HTN medications and among them, 78 (37.6%) were on one drug while only 14 (6.8%) were using four drugs to control their BP. When we observed the type of anti-HTN drugs, we noted that the majority of the patients were taking beta-blockers (162; 78.3%) from which 112 (69.1%) were using dialyzable, while 50 (30.9%) were on a non-dialyzable beta-blocker (Table 2).

**Table 2:** Antihypertensive medication status of the patients (n=207)

Antihypertensive medication status of the patients n (%)		
Patients on antihypertensive medications		207(78.7)
No of antihypertensive(s)	1	78(37.6)
	2	75(36.2)
	3	40(19.3)
	4	14(6.8)
Type of antihypertensives	Calcium channel blocker	135(65.2)
	Beta-blockers	162(78.3)
	Dialyzable beta blocker (n=162)	112(69.1))
	Non-dialyzable beta blocker (n=162)	50(30.9)
	Vasodilator	54(26.1)
	Angiotensin receptor blocker	34(16.4)
	Alpha blocker	7(3.4)
	Angiotensin-converting enzyme inhibitor	4(1.9)
Compliance of patients with antihypertensive medication		177(85.5)

We observed that the mean of pre- and post-hemodialysis BP was statistically the same in our patients (p>0.05) (Table 3). The mean interdialytic weight gain (IDWG) of the study population was 1.76 (±0.82) Kg, that is equivalent to 2.82 (±1.43) % of dry weight.

**Table 3:** Mean differences of pre- and post-hemodialysis blood pressure (BP) of the patients

Parameters	Mean ± STD	Minimum	Maximum	P value	
Pre hemodialysis systolic BP	141.1 ± 16.8	86.5	186	0.606	
Post hemodialysis systolic BP	140.7 ± 15.9	91.8	180		
Pre hemodialysis diastolic BP	76.2 ± 10.7	46.8	105.5	0.243	
Post hemodialysis diastolic BP	75.6 ± 8.1	51.3	100.2		

The overall prevalence of intradialytic hypertension was 16% among our participants in the study and we noted that the age of the patients was significantly associated with intradialytic hypertension (p=0.038). We observed that the mean age was higher in patients who had intradialytic HTN as compared to the patients who did not have it (55.3±14.3 v/s 50.2±14 years respectively). On the other hand, gender and all comorbid conditions were equally distributed in both groups of patients (p>0.05) (Table 4).

**Table 4:** Association of demographic and clinical parameters with Intradialytic hypertension n (%) / Mean ± STD

Demographic and comorbid variables		Intradialytic hypertension		p-value
		No =221(84%)	Yes =42(16%)	
Age		50.2 ± 14	55.3 ± 14.3	0.038
Gender	Male	127(57.5)	21(50)	0.371
	Female	94(42.5)	21(50)	
Diabetes mellitus	No	140(63.3)	27(64.3)	0.908
	Yes	81(36.7)	15(35.7)	
Hypertension	No	27(12.2)	3(7.1)	0.343
	Yes	194(87.8)	39(92.9)	
Ischemic heart disease	No	175(79.2)	31(73.8)	0.438
	Yes	46(20.8)	11(26.3)	
Cardiomyopathy	No	176(79.6)	33(78.6)	0.875
	Yes	45(20.4)	9(21.4)	
Cardiovascular disease	No	201(91)	38(90.5)	0.922
	Yes	20(2)	4(9.5)	
Hepatitis B +ve	No	207(93.7)	38(90.5)	0.453
	Yes	4(6.3)	4(9.5)	
Hepatitis C +ve	No	209(94.6)	39(92.9)	0.661
	Yes	12(5.4)	3(7.1)	
Transplant rejection	No	217(98.2)	39(92.9)	0.084

The majority of the patients with IDH were those who were taking anti-HTN medications as compared to the patients who were not taking them (p<0.004). On the contrary, the difference in mean of pre- and post-dialytic weight gain was higher in patients without intradialytic HTN than the patients with it (1.8±0.7 v/s 1.7±1.3 respectively) (Table 5).

**Table 5:** Association of antihypertensive medications, dialysis vintage, and mean interdialytic weight gain (IDWG) with intradialytic hypertension n (%) / Mean ± STD

Variables		Intradialytic hypertension		P value	
		No =221(84%)	Yes =42(16%)		
Number of antihypertensive drugs	No	54(24.4)	2(4.8)	0.004	
	1	65(29.4)	13(31)		
	2	62(28.1)	13(31)		
	3	27(12.2)	13(31)		
	4	13(5.9)	1(2.4)		
Non-dialyzable beta blocker	No	179(81)	34(81)	0.995	
	Yes	42(19)	8(19)		
Dialyzable beta blocker	No	129(58.4)	22(52.4)	0.472	
	Yes	92(41.6)	20(47.6)		
Dialysis vintage	3 - 6 months	10(4.5)	4(9.5)	0.644	
	6.1 - 12 months	23(10.4)	3(7.1)		
	1.1 - 3 years	66(29.9)	15(35.7)		
	3.1 - 5 years	40(18.1)	6(14.3)		
	5.1 - 10 years	56(25.3)	11(26.2)		
	> 10 years	26(11.8)	3(7.1)		
Average intradialytic weight gain		1.8 ± 0.7	1.7 ± 1.3	0.009	
Percentage of Intradialytic weight gain		2.8 ± 1.3	2.8 ± 2.1	0.12	
Dialysate calcium	2	20(9)	2(4.8)	0.358	
	3	201(91)	40(95.2)		

We made a multivariate predictive model for the IDH by adding all significant predictors of univariate analysis and found that average intradialytic weight gain became insignificant in this model, while age and use of anti-HTN medications remained the significant predictors for IDH. We perceived that a one-year increase in age can cause a 3% increase in intradialytic HTN (odds ratio 1.03 CI 1.006-1.06 p=0.018). Similarly, the patients who were taking one anti-HTN drug had a 5.5 times more chance to develop intradialytic HTN as compared to the patients who were not taking any medication (odds ratio 5.5 CI 1.2-25.7 p=0.031). Same as the patients who were using three anti-HTN were 15.8 times more prone to intradialytic HTN than no use of medication (odds ratio 15.8 CI 3.2-76.9 p=0.001) (Table 6).

**Table 6:** Multivariate logistic regression model showing the amount of effect of independent variables on the presence of intradialytic hypertension

Variables		Odds ratio	95% CI		P value	
			Lower	Upper		
Age		1.03	1.006	1.06	0.018	
Average Intradialytic weight gain		0.8	0.5	1.3	0.36	
Number of antihypertensive drugs	No use	1				
	1 drug	5.5	1.2	25.7	0.031	
	2 drugs	5.6	1.2	26.4	0.028	
	3 drugs	15.8	3.2	76.9	0.001	
	4 drugs	2.1	0.17	25.1	0.564	

## Discussion

Our study identified the frequency of intradialytic hypertension among Pakistani adults on MHD as per the latest KDIGO suggested definition. This would add vital evidence to the existing literature of our country and the rest of the world as the frequency of IDH as per KDIGO's suggested definition has not been investigated much. Most of the data is based on earlier definitions in use. Intradialytic hypertension reported in our study was found among 16% of the participants. Our study has reported relatively low estimates of IDH compared to other studies, which have reported an estimated frequency of IDH ranging from 22.3% to 44.5% according to the various definitions used earlier [9-12].

The diagnosis and management of hypertension in patients on maintenance hemodialysis depend on the measurement of BP from the pre- to post-dialysis period as patients with intradialytic hypertension are found to be chronically volume overloaded as compared to other hemodialysis patients [15]. For such purpose, clinicians must follow some standard guidelines and measures with the aim of a consistent approach to assess IDH and make every effort to develop consensus for best practices in management and treatment modalities [2]. Established research examined the association of different definitions to measure intradialytic hypertension with all-cause mortality and the findings suggested that patients with an increase in SBP from pre- to post-hemodialysis faced the highest risk of mortality [16]. While it is a fact that IDH has been associated with an increased risk of mortality, its association with increased vascular resistance during the dialysis period has been poorly explained and is overshadowed by its consistent link with volume overload leading to aggressive fluid management as an initial management option [1]. Therefore, ambiguity in understanding the mechanism and identification of a clear approach to diagnosing IDH leaves a broader area to be explored for dialysis management. 

In our study age of the patients was significantly associated with IDH (p=0.038). Studies have shown a consistent association of IDH with old age and multiple comorbidities [17]. The major comorbidities found among our study participants were hypertension in 88.6%, diabetes mellitus in 36.5%, ischemic heart disease in 21.7%, and cardiomyopathy and cerebrovascular disease in 20.5% and 9.1%, respectively. Various clinical outcomes as a result of IDH have been reported in previous studies such as metabolic disorders, all-cause mortality, volume-associated hospitalization, deaths due to cardiac failure, and hospitalization associated with cardiovascular disease [18,19].

Our study reported that 91.6% of patients received dialysate calcium 3mEq/L and only 8.4% received dialysate calcium 2mEq/L. Most patients at our facility have low calcium and high parathyroid hormone, therefore, dialysate calcium is kept at 3mEq/L to prevent hyperparathyroidism. This rationale has been supported by KDIGO CKD-MBD guidelines of 2017 which advocate the use of 2.5-3mEq/L dialysate calcium to lower serum phosphorus and maintain serum calcium [20]. Recent studies have shown that low dialysate calcium can be used to treat IDH, however this is at the cost of increased risk for cardiac arrhythmias and negative calcium balance [21].

Antihypertensive medications were used by 78.7% of patients, out of which 85.5% of the patients were compliant with antihypertensive(s). Compliance was checked by interviewing the patients. This method of checking compliance may have recall and response bias. It was not possible to check their pharmacy record as many of them bought medicines from outside our facility. Single or dual antihypertensive agents were prescribed to 37.6% and 36.2% of patients respectively. 19.3% and 6.8% of patients were prescribed three and four types of antihypertensive agents which has depicted a serious effort to control persistently elevated intradialytic hypertension. Beta-blockers were the most used antihypertensive medication in 78.3% of patients, followed by calcium channel blockers prescribed to 65.2% of patients. Vasodilators and angiotensin receptor blockers were prescribed to 26.1% and 16.4% of patients, respectively. Very few patients were using alpha-blocker and angiotensin-converting enzyme inhibitors, 3.4% and 1.9% respectively. No recommendations are available for any specific antihypertensive medication class due to the heterogeneity of patient characteristics and lack of comparative evidence. Medication selection should be based on cardiovascular indications, patient characteristics, and side effect profile of drugs [2]. Flythe et al. mentioned that the first-line antihypertensive drugs (e.g. angiotensin receptor blockers or angiotensin-converting enzyme inhibitors, and calcium channel blockers) recommended for the general population can be suggested to patients on dialysis as first-line antihypertensives [2].

Out of 162 patients on beta-blockers, 69.1% were on dialyzable beta-blockers like metoprolol, atenolol and bisoprolol while 30.9% were on non-dialyzable drugs like carvedilol, propranolol and nebivolol. In contrast to earlier research [19] our study did not find an association of IDH with dialyzable beta-blockers. Important parameters to be considered in the selection of antihypertensives are dialyzability and pharmacokinetics. Findings from a retrospective study have identified that beta-blockers with a high dialyzability such as atenolol and metoprolol were associated with a high risk of mortality than beta-blockers with a low dialyzability such as bisoprolol and propranolol [22]. Another retrospective cohort study, in contrast, reported higher mortality rates in patients who received non-dialyzable carvedilol which attributed to a higher probability of intradialytic hypotension compared to the highly dialyzable beta-blocker such as metoprolol [23]. In addition to these controversial findings, uncertainties have been observed in the data measuring the drug dialyzability of hi-flux dialysis as a recent study finding suggested that bisoprolol contrary to its previous features might be dialyzable, therefore, intradialytic BP patterns should be considered with drug dialyzability [24].

Flythe et al. suggested that the use of long-acting and once-daily medication with relatively stable intradialytic blood pressure might help improve adherence and reduce the pill burden [2]. The administration of the antihypertensive drug should be timed according to individual characteristics, considering the intradialytic hypotension frequency and interdialytic BP [2].

Among patients with IDH, 30.8% were dialysis dependent for 1.1 to three years while 25.5% were dialysis dependent for 5.1 to 10 years. Earlier studies have shown an association between IDH with shorter dialysis duration [1]. However, our study shows no association of IDH with dialysis vintage. The mean age of patients with IDH was higher than those without IDH in our study. Moreover, patients who were taking one anti-HTN drug had 5.5 times more chance to develop IDH in comparison to the patients who were not taking any medication, while patients who were using three anti-HTN were 15.8 times more prone to IDH than those not using any anti-hypertensive. Our findings are consistent with the CLIMB study in which patients with IDH were older, used more antihypertensive drugs and had lower dry weight and interdialytic weight gain [8]. The mean IDWG of our study population was 1.76 (±0.82) Kg, which is equivalent to 2.82 (±1.43)% of dry weight. Volume overload was not a consistent finding in our patients with IDH which suggests other etiological factors as described in earlier research [3,5-7]. A multi-pronged approach is needed to manage IDH such as adjustment of dialysate, re-evaluation of dry weight, the adaptation of appropriate blood pressure measurement methods, and adjustment of antihypertensive medications, all of which have been required among IDH patients who remain at high risk of mortality [17].

Our study possesses strengths in highlighting the importance of standard criteria to diagnose IDH and gathering important facts that will add value to existing literature. A few limitations of our study are that it is a single-centre study, therefore our findings lack generalizability. Since this was a cross-sectional study the temporal association could not be explored. The method of checking compliance may have recall and response bias.

## Conclusions

In conclusion, the frequency of IDH in our study according to the latest KDIGO definition is much lower than frequencies calculated from earlier definitions. Various definitions to label IDH can lead to variation in the estimation resulting in diverse approaches in IDH case management. With a high risk of morbidity and mortality in association with IDH, there is an imminent need to reach a unified consensus on the definition of IDH and the establishment of best practice guidelines accordingly. The association of fluid overload with IDH was also not found in this study which suggests the need for evaluation of other etiological factors.
